# Imp and Syp RNA-binding proteins govern decommissioning of *Drosophila* neural stem cells

**DOI:** 10.1242/dev.149500

**Published:** 2017-10-01

**Authors:** Ching-Po Yang, Tamsin J. Samuels, Yaling Huang, Lu Yang, David Ish-Horowicz, Ilan Davis, Tzumin Lee

**Affiliations:** 1 Howard Hughes Medical Institute, Janelia Research Campus, 19700 Helix Drive, Ashburn, VA 20147, USA; 2 Department of Biochemistry, University of Oxford, Oxford OX1 3QU, United Kingdom; 3 MRC Laboratory for Molecular Cell Biology, University College London, London WC1E 6BT, United Kingdom

**Keywords:** Neuroblast, RNA-binding protein, Mediator complex, Cell cycle exit, Mushroom body

## Abstract

The termination of the proliferation of *Drosophila* neural stem cells, also known as neuroblasts (NBs), requires a ‘decommissioning’ phase that is controlled in a lineage-specific manner. Most NBs, with the exception of those of the mushroom body (MB), are decommissioned by the ecdysone receptor and mediator complex, causing them to shrink during metamorphosis, followed by nuclear accumulation of Prospero and cell cycle exit. Here, we demonstrate that the levels of Imp and Syp RNA-binding proteins regulate NB decommissioning. Descending Imp and ascending Syp expression have been shown to regulate neuronal temporal fate. We show that Imp levels decline slower in the MB than in other central brain NBs. MB NBs continue to express Imp into pupation, and the presence of Imp prevents decommissioning partly by inhibiting the mediator complex. Late-larval induction of transgenic Imp prevents many non-MB NBs from decommissioning in early pupae. Moreover, the presence of abundant Syp in aged NBs permits Prospero accumulation that, in turn, promotes cell cycle exit. Together, our results reveal that progeny temporal fate and progenitor decommissioning are co-regulated in protracted neuronal lineages.

## Abstract (precis)

**Highlighted Article:** Temporal progression of Imp/Syp gradients determines the timing of neuroblast decommissioning and cell cycle exit in addition to progeny temporal fate, allowing proper completion of a neuronal lineage.

## INTRODUCTION

A long-lived neural stem cell shows age-dependent changes in proliferation and progeny cell fate (Li et al., 2013). In *Drosophila*, temporal regulation is lineage specific. Individual neuroblasts (NBs) produce a unique series of neuronal types and end neurogenesis at independent times in development (Chai et al., 2013; Homem et al., 2015; Lee et al., 1999; Lin et al., 2012; Sousa-Nunes, et al., 2010; Truman and Bate, 1988; Yu et al., 2010). The timing of termination of individual NBs is likely to be co-regulated with their temporal fate, which guides birth order-dependent neuronal identities, to produce the required number and types of neurons in each lineage (Maurange et al., 2008). Such co-regulation could be achieved by a lineage-autonomous developmental clock that governs both progeny temporal fate and NB aging in each neuronal lineage.

Temporal protein gradients, including IGF-II mRNA-binding protein (Imp), Syncrip/hnRNPQ (Syp) and Chronologically inappropriate morphogenesis (Chinmo) govern neuronal temporal fates in diverse NB lineages (Liu et al., 2015; Ren et al., 2017; Zhu et al., 2006). The RNA-binding proteins Imp and Syp are expressed in the NB, with Imp levels descending and Syp levels ascending over time. The serially derived neuronal progeny inherit the Imp and Syp levels from the NB. Imp enhances and Syp represses the translation of Chinmo, a BTB zinc-finger nuclear protein. This results in a high-to-low Chinmo protein gradient among the newborn neurons. The opposing Imp/Syp temporal gradients (as revealed by RNA-seq) extend throughout neurogenesis but exhibit distinct lineage-characteristic expression levels and progression rates (Liu et al., 2015; Ren et al., 2017). In the antennal lobe (AL) lineages, one NB can yield a sequence of more than 20 neuronal types from ∼80 asymmetric cell divisions that span over 4 days of larval development (Yu et al., 2010; Lin et al., 2012). The opposing Imp/Syp temporal gradients are steep and progress rapidly in such fast changing NBs. By contrast, the NBs that make neurons of the mushroom body (MB) learning and memory center continue to divide throughout larval and pupal development but produce only three morphological classes of neurons (Lee et al., 1999). Imp and Syp are expressed in shallow, slowly progressing gradients in the long-lived MB NBs. The close correlation between the progression of Imp/Syp gradients and the progeny's temporal fate changes argues for direct coding of diverse neuronal temporal fates by distinct levels of Imp and/or Syp.

NB termination is also temporally regulated. During active cycling, NBs regrow promptly following each asymmetric cell division (Knoblich, 2008; San-Juán and Baonza, 2011; Song and Lu, 2011). Upon exposure to a pulse of ecdysone hormone, all NBs, except the four pairs of MB NBs, undergo size reduction while cycling because of sluggish regrowth. The size-reduced NBs divide slowly and eventually divide symmetrically into two post-mitotic cells (Homem et al., 2014; Maurange et al., 2008). The stage-specific progressive termination of NBs is an actively regulated process, which we refer to as ‘decommissioning’.

The initiation of NB decommissioning depends on the ecdysone receptor and core components of the mediator complex, which promote key enzymes to increase oxidative phosphorylation in energy metabolism that slows down NB regrowth (Homem et al., 2014). Subsequent nuclear accumulation of Prospero then drives cell cycle exit of NBs (Maurange et al., 2008). Individual NBs exit cell cycle at characteristic times around 24 h after pupal formation (APF) (Awasaki et al., 2014). However, the mechanisms underlying such temporal differences in NB decommissioning remain unexplored. Moreover, despite global ecdysone action, the MB NBs actively regrow and cycle for ∼100 more divisions at the pupal stage (Ito and Hotta, 1992; Truman and Bate, 1988). The MB NBs finally shrink but terminate by apoptosis around 96 h APF (Siegrist et al., 2010).

Given that individual lineages vary in length and produce diverse neuron types in distinct time courses, it is likely that NB decommissioning and temporal fate progression are co-regulated in time. However, it is not known what mechanisms are responsible for such co-regulation or what factors are involved. Here, we address this question by examining whether and how the temporal factors Imp and Syp guide the course of NB decommissioning. We found that Imp prevents MB NBs from shrinkage in early pupae partly through inhibiting the mediator complex. Other NBs lacking Imp shrink rapidly in early pupae, and exit the cell cycle upon Syp-dependent nuclear accumulation of Pros. Imp is dominant to Syp, as Syp can promote cell cycle exit only in Imp-negative decommissioned NBs. Taken together, NB decommissioning is an actively programmed developmental process that is temporally regulated by the levels of Imp and Syp proteins in each NB to ensure the proper completion of a neuronal lineage.

## RESULTS

### Imp and Syp play independent roles in NB decommissioning

**Fig. 1.**
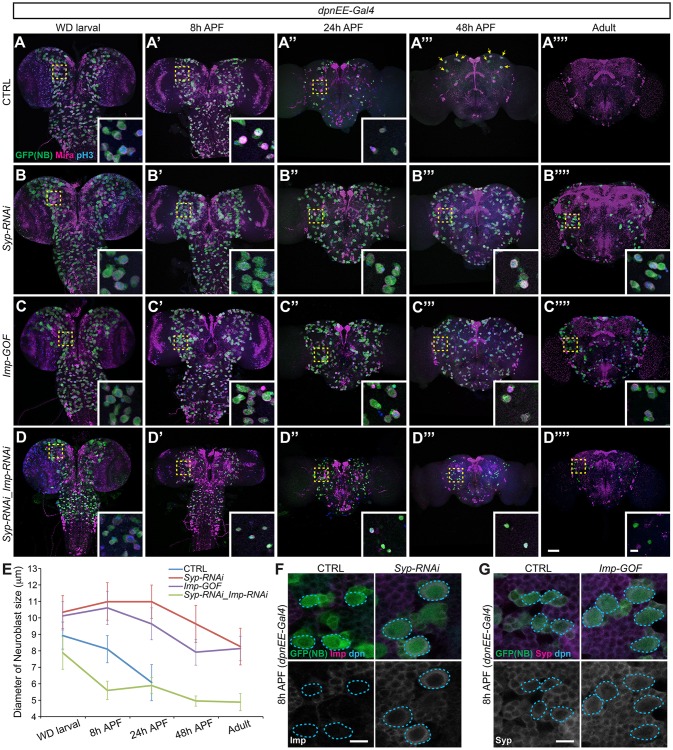
**Imp and Syp regulate non-MB NB decommissioning.** (A-D″″) Ectopic Imp or Syp-depletion prolongs NB life into the adult. Composite confocal images of control (CTRL; A-A″″), *Syp* RNAi (B-B″″), *Imp* gain of function (GOF; C-C″″), and *Syp/Imp*-depleted (D-D″″) fly brains at specific developmental times, immunostained for GFP (green), Mira (magenta) and phospho-Histone H3 (pH3, blue). Transgenes were driven by *dpnEE-GAL4* in the NBs of central brain. Yellow arrows indicate the MB NBs. Insets show the boxed areas at higher magnification. Scale bar: 50 µm (10 µm in inset). (E) Quantification of NB size in the anterior region of the fly brain (measured by the diameter of Mira-labeled NBs, mean±s.d., *n*=6 brains). (F) *Syp* depletion prolongs NB Imp expression. Representative confocal images of 8 h APF fly brains immunostained for Imp (magenta), GFP (green) and Dpn (blue) in control and *Syp* depletion conditions/experiments driven by *dpnEE-Gal4*. Scale bar: 10 µm. (G) *Imp* gain of function did not affect Syp expression. Representative confocal images of 8 h APF fly brains immunostained for Syp (magenta), GFP (green) and Dpn (blue) in control and *dpnEE-Gal4*-driven *Imp* gain-of-function conditions/experiments. In F and G, NBs with a maximum diameter at the given focal plane are circled. Scale bar: 10 µm.

In order to monitor decommissioning of central brain NBs, we expressed a cell cycle-sensitive GFP using a GAL4 driver that is active in all but optic lobe NBs (Fig. S1B). In the wild-type control, almost all NBs had disappeared by 48 h APF, except for four remaining MB NBs, previously described as actively dividing until 96 h APF (Siegrist et al., 2010). As expected, the MB NBs had disappeared by adult stage (Fig. 1A″″). We followed the progressive ending of non-MB NBs (∼100 per hemisphere) from wandering larvae to early pupae. Their average size had decreased significantly by 8 h APF, and mostly reduced to a size comparable to that of their daughter ganglion mother cells (GMCs) within 24 h APF (Fig. 1A-A″,E). Notably, the aged NBs did not exit the cell cycle in a synchronized wave. A few NBs (though small in size) remained at 24 h APF (Fig. 1A″), as evidenced by the expression of Miranda (Mira), an NB-specific gene (Ikeshima-Kataoka et al., 1997), and the occasional presence of phospho-Histone3 (pH3), indicating mitosis (Fig. S1A).

Those progressively ending NBs in early pupae were negative for Imp and positive for Syp (Fig. 1F,G, Fig. S2B,C). Most, if not all, NBs show abundant Imp and minimal Syp in early larvae (Fig. S2A-C). We therefore wondered if NBs purposely locked in the initial state of Imp/Syp expression (high Imp, low to no Syp) could escape decommissioning. We tested this idea by silencing Syp with targeted RNAi, which consequently maintained detectable Imp throughout NB life (Fig. 1F, Fig. S2B). We found that NBs with persistent Imp and minimal Syp expressions escaped decommissioning (Fig. 1B). Most, if not all, NBs remained at 48 h APF (Fig. 1B-B‴); a few sustained and continued to cycle at the adult stage (Fig. 1B″″, Fig. S1). Moreover, the size of Syp-depleted NBs was not reduced by 24 h APF, and those that persisted were consistently larger than GMCs (Fig. 1E). Continuously expressing transgenic Imp elicited similar phenotypes (Fig. 1C-C″″). We examined the altered Imp/Syp levels by immunostaining (Fig. S2). Notably, Imp/Syp mutual inhibition is less evident with overexpression experiments than with RNAi depletion. Hence, levels of Syp remained relatively high in Imp-overexpressing NBs in early pupae that showed no evidence of ageing (Fig. 1G, Fig. S2C). This result argues that it is ectopic Imp, rather than the absence of Syp, which accounts for the suppression of early pupal NB decommissioning in both loss-of-Syp and gain-of-Imp conditions.

Consistent with Imp dominantly repressing NB decommissioning, silencing Imp together with Syp restored the early-pupal NB shrinking (Fig. 1D-D″″). NBs with co-depleted Imp and Syp underwent accelerated shrinkage in early pupae, indicating rapid ageing in response to the ecdysone- and mediator-mediated metabolic change (Fig. 1E). However, many of the NBs that shrank failed to terminate until late pupal or even adult stage (Fig. 1D″″, Fig. S1). Taken together, our data suggest that Imp levels determine whether NBs shrink in early pupae. Once decreased in size, the NBs require Syp to exit the cell cycle.

### MB NBs escape early pupal decommissioning owing to protracted Imp expression

**Fig. 2.**
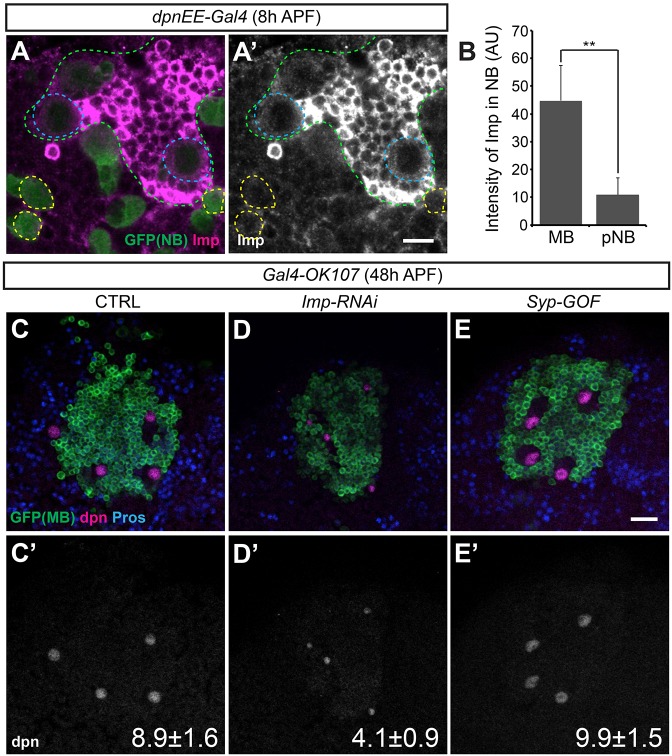
**Protracted Imp expression protects MB NBs from early pupal decommissioning.** (A) Imp is continuously expressed in MB NBs at early pupal development. Representative confocal images of 8 h APF wild-type fly brain immunostained for GFP (green) and Imp (magenta). The green dashed line indicates the MB region (note high Imp levels); MB NBs (circled with blue dashed line) show protracted Imp expression. The yellow dashed line circles non-MB NBs (posterior NB, pNB) at the same focal plane, which are negative for Imp expression. Scale bar: 10 µm. (B) Quantification of the grayscale value for Imp immunostaining in the MB NBs and pNBs in 8 h APF wild-type flies. **P<0.01 (Student's *t*-test) (mean±s.d., *n*=6 brains). AU, arbitrary fluorescent intensity units. (C-E′) *Imp* depletion prematurely ended MB neurogenesis. Representative confocal images of 48 h APF fly brains immunostained for GFP (green), Dpn (magenta) and Pros (blue) in control (CTRL; C,C′), *Imp* depletion (D,D′) and *Syp* gain of function (GOF; E,E′) experiments/conditions. Transgenes were driven by *GAL4-OK107*; diameter of MB neuroblast at 48 h APF (mean±s.d., *n*=6 brains) are indicated at the bottom right of each panel. Scale bar: 20 µm.

At the late larval stage, only the MB NBs maintain detectable levels of Imp (Fig. 2A-B). We therefore tested whether Imp expression in the MB NBs is responsible for their long life. Indeed, targeted RNAi rendered Imp undetectable in larval MB NBs (data not shown) and resulted in a premature stop of MB neurogenesis in early pupae. Without Imp, the MB NBs were relatively small but stable in size until pupation when they rapidly shrank (Fig. 5E). The majority of Imp-depleted MB NBs survived beyond 48 h APF [3.5±0.8 (mean±s.d.) per brain lobe in Imp RNAi versus 4.0±0 in wild-type control], but had a drastically reduced cell size (Fig. 2D,D′ compared with 2C,C′) and were never found to be positive for pH3 (data not shown).

Wild-type MB NBs also express a much lower level of Syp than non-MB NBs (Liu et al., 2015; Ren et al., 2017). We therefore tested whether the lower Syp levels were responsible for the extended lives of MB NBs. Overexpressing Syp in MB NBs did not cause these cells to undergo decommissioning in early pupae (Fig. 2E,E′). Even using a stronger pan-NB driver to induce Syp (either one or two copies) did not result in premature MB NB shrinking (Fig. S3). Taken together, our results indicate that prolonged Imp expression, rather than lower Syp expression, protects MB NBs from decommissioning in the early pupa.

### Late-larval induction of Imp effectively protects non-MB NBs from early pupal decommissioning

It is possible that MB and non-MB NBs undergo progressive ageing at different speeds during larval development and only the non-MB NBs have aged sufficiently to undergo decommissioning around pupal formation. If this is true, overexpressing Imp from the beginning of neurogenesis could slow down the ageing of NBs and indirectly affect their responses to ecdysone. Alternatively, Imp might acutely regulate NB decommissioning by governing ecdysone signaling. To address this possibility, we tried not to perturb normal NB ageing (if any) and examined whether late larval induction of transgenic Imp could protect non-MB NBs from early pupal decommissioning.

**Fig. 3.**
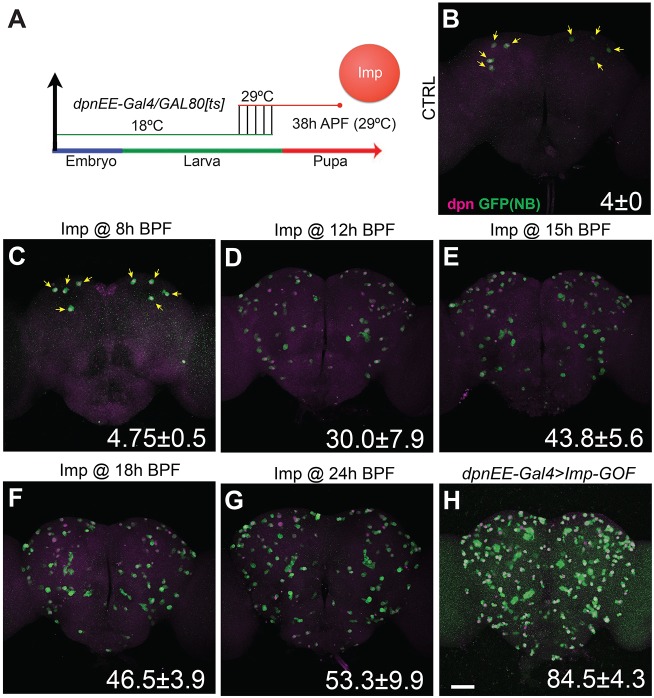
**Induction of transgenic Imp at late larval stage prolongs NB life.** (A) Scheme for induction time course of transgenic Imp. Heat shock inactivated the temperature-sensitive GAL80 at specific developmental times, allowing *dpnEE-GAL4* to drive Imp expression. Flies were continuously incubated at 29°C after heat shock and fly brains were processed 38 h after pupal formation (38 h APF). (B-H) Late larval induction of Imp prolongs NB life. Composite confocal images of fly brains, immunostained for GFP (green) and Dpn (magenta). Experimental conditions were control (CTRL; B), targeted transgenic Imp induction at 8 h (C), 12 h (D), 15 h (E), 18 h (F) and 24 h (G) before pupal formation (BPF), and flies lacking GAL80[ts], which allowed continuous Imp expression (H). Yellow arrows indicate the MB NBs. The number of NBs per brain lobe (mean±s.d., *n*=6) is indicated at the bottom right of each panel. Scale bar: 50 µm.

We temporally controlled the induction of transgenic Imp in all NBs positive for *dpnEE-GAL4* (Awasaki et al., 2014), using a temperature-sensitive GAL80 (McGuire, et al., 2003). Transgenic Imp was induced at a specific time around pupation with a temperature shift from 18°C to 29°C (Fig. 3A). Induction at 8 h before pupa formation (BPF) or later failed to extend the life of non-MB NBs beyond early pupal development (Fig. 3C; data not shown). Induction at 12 h and 15 h BPF, by contrast, substantially prolonged the life of 30% and 44% of non-MB NBs, respectively (Fig. 3D,E). Induction earlier than 15 h BPF only increased the percentage of sustained NBs to ∼50% (Fig. 3F,G) as opposed to ∼85% with continuous Imp expression (Fig. 3H). The incomplete penetrance was probably due to a mosaic inactivation of GAL80[ts], as a comparable percentage (∼65%) of NBs had detectable Imp levels and expression of a UAS reporter approximately 12 h after induction (data not shown). Continuous co-induction of Imp and an anti-apoptotic p35 transgene did not extend more NBs into the adult stage (Fig. S4). This result argues against the possibility that Imp expression selectively prolongs NBs that end by cell cycle exit rather than apoptosis. The potency of late larval Imp induction in sustaining many non-MB NBs suggests that Imp can act acutely to suppress NB decommissioning during the larval-to-pupal transition.

### Imp suppresses NB decommissioning partly by inhibiting the mediator complex

The ecdysone receptor and the mediator complex have been recently implicated in NB shrinking and ultimately ending (Homem et al., 2014). The mediator complex is essential for a switch in energy metabolism that slows down NB regrowth during successive divisions and thus progressively reduces the NB cell size. NB shrinkage and termination can also be delayed by expressing Med12 (Kohtalo, Kto), the inhibitory subunit of the mediator complex (Homem et al., 2014; Malik and Roeder, 2010).

**Fig. 4.**
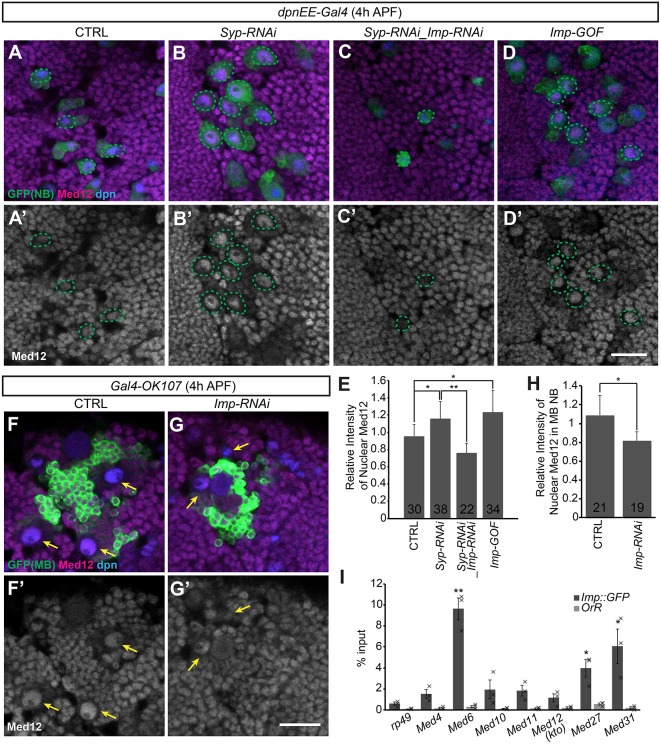
**Imp interacts with mediator complex.** (A-D′) Imp promotes Med12 expression in NBs. Representative confocal images of 4 h APF fly brains immunostained for GFP (green), Med12 (magenta) and Dpn (blue) in control (CTRL; A,A′), *Syp* RNAi (B,B′), *Syp/Imp* depletion (C,C′) and *Imp* gain of function (D,D′) driven by *dpnEE-Gal4*. Green dashed lines circle NBs with a maximum diameter at the given focal plane. Scale bar: 20 µm. (E) Quantification of the relative intensity of Med12 immunostaining in NBs of the anterior region of fly brains as shown in A-D (mean±s.d., number of sampled NBs is indicated for each experiments/condition from 6 brains). *P<0.05, **P<0.01 (Student's *t*-test). (F-G′) Imp promotes Med12 expression in MB NBs. Representative confocal images of 4 h APF fly brains immunostained for GFP (green), Med12 (magenta) and Dpn (blue) in control (CTRL; E) and after *Imp* depletion (F) driven by *Gal4-OK107*. Arrows indicate the MB NBs. Scale bar: 20 µm. (H) Quantification of Med12 immunostaining in the MB NBs (relative to optic lobe) as shown in F and G. *P<0.05 (Student's *t*-test) (mean±s.d., number of sampled NBs is indicated for each experiment/condition from 6 brains). (I) *Med6*, *Med27* and *Med31* mRNAs could be effectively pulled down with Imp. RT-qPCR (mean±s.e.m., *n*=3) of RNA immunoprecipitation from *Imp::GFP* and *Oregon-R* larval brain lysates shows the percent inputs of candidate mediator complex components that were recovered by anti-GFP immunoprecipitation. Note that *Med12* was not significantly enriched. *P<0.05, **P<0.01 (Student's *t*-test comparing the recovery of each transcript to that of the non-binding *rp49*).

To examine the possibility that Imp prevents NB shrinkage by suppressing the mediator complex, we determined Med12 expression in the NBs following various Imp/Syp manipulations. We found elevated Med12 protein levels around pupation in non-MB NBs with Syp RNAi and Imp overexpression (Fig. 4A-B′,D,D′,E). The Med12 protein levels were reduced to lower than normal upon silencing both Imp and Syp using RNAi (Fig. 4C,C′,E). Imp-depleted MB NBs also showed a significant reduction in Med12 expression (Fig. 4F-H). These observations suggest that Imp promotes Med12, which could in turn suppress the mediator complex and thus prevent NB shrinkage.

To test whether the RNA-binding protein Imp directly associates with transcripts encoding Med12 and/or other mediator complex subunits, we pulled down endogenous Imp::GFP (Toledano, et al., 2012) and quantified various co-precipitated transcripts by qPCR following RNA immunoprecipitation (RIP-qPCR). Eight mediator complex components, including Med4, Med6, Med9, Med10, Med11, Med22, Med27 and Med31, have been implicated in promoting NB shrinkage following pupation (Homem et al., 2014). Among them, *Med6*, *Med27* and *Med31* transcripts were significantly enriched over the negative control *rp49* (*RpL32*) (Fig. 4I). We confirmed the requirement of Med6 for prompt NB shrinking by RNAi. Moreover, silencing Med6 could effectively block the accelerated NB shrinkage caused by Imp RNAi, as double RNAi (like repressing Med6 alone) delayed NB shrinkage and sustained most NBs into 48 h APF (Fig. S5). By contrast, the *Med12* mRNA could not be significantly enriched (Fig. 4I). In all cases, the control GFP immunoprecipitation from a wild-type strain was very clean, ranging from 0.09% to 0.54% of input (Fig. 4I). These results implicate multiple core components of the mediator complex, but not Med12, as Imp direct targets.

**Fig. 5.**
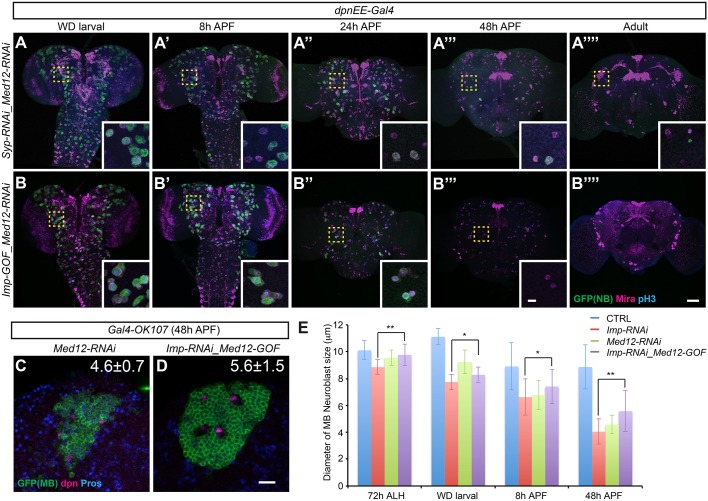
**The mediator complex functions downstream of Imp to induce NB shrinkage.** (A-B″″) Med12 depletion blocks the inhibition of NB shrinkage caused by persistent Imp expression. Composite confocal images of *Syp* and *Med12* joint knockdown (A) and *Imp* gain of function combined with *Med12* knockdown (B) driven by *dpnEE-Gal4.* Brains were dissected at specific developmental ages and immunostained for GFP (green), Mira (magenta) and phospho-Histone H3 (pH3, blue). Insets show the boxed areas at higher magnification. Scale bar: 50 µm (10 µm in inset). (C-E) The mediator complex acts downstream of Imp to regulate MB NB shrinkage. Representative confocal images of 48 h APF fly brains immunostained for GFP (green), Dpn (magenta) and Pros (blue) in *Med12* depleted (C) and combined *Imp*-depleted with *Med12* gain-of-function conditions (D) driven by *Gal4-OK107*. Diameter of MB NBs at 48 h APF (mean±s.d., *n*=6 brains) is indicated at the top right of each panel. Scale bar: 20 µm. (E) Mean MB NB diameter (±s.d., *n*=6 brains) of flies at different developmental stages with transgenic manipulations driven by *GAL4-OK107*. *P<0.05, **P<0.01 (Student's *t*-test).

Although *Med12* was not recovered as a direct target of Imp, an easy way to modify the mediator complex's activity is by knocking down or overexpressing the inhibitory subunit Med12 (Malik and Roeder, 2010). To test this possibility, we examined the genetic interactions between Imp/Syp and the mediator complex by manipulating Med12. Syp-depleted NBs did not shrink normally (Fig. 1B). However, addition of Med12 RNAi, which activates the mediator complex, rescued the early pupal NB shrinking back to wild type (Fig. 5A-A″″). The size-reduced NBs (positive for Mira despite a gradual loss of labeling with *dpnEE-GAL4*) did not exit the cell cycle; instead they persisted with evidence of cycling into adult stage (Fig. 5A″″, Fig. S1A). This is reminiscent of the tiny non-MB NBs caused by repression of both Imp and Syp (Fig. 1D″″). By contrast, activating the mediator complex by silencing Med12 could make Imp-overexpressing NBs not only shrink but also exit the cell cycle by 48 h APF (Fig. 5B-B″″). Further, silencing Med12 alone was also sufficient to make MB NBs shrink rapidly in early pupae, similar to non-MB NBs (Fig. 5C). These results suggest that Imp suppresses NB decommissioning by inhibiting the mediator complex.

However, overexpressing Med12 only partially blocked the rapid shrinkage of Imp-depleted MB NBs in early pupae (Fig. 5D with an average diameter of 5.6±1.5 at 48 h APF, compared with Fig. 2D′ with 4.1±0.9 in Imp RNAi and Fig. 2C′ with 8.9±1.6 in wild-type control). Furthermore, silencing Med6 failed to protect non-MB NBs beyond the mid-pupal stage (Fig. S5A-A‴). These limited effects, compared with Imp overexpression that could sustain many NBs into adult stage, argue that Imp plays additional roles in suppression of NB shrinkage. Taken together, we can conclude that Imp prevents NB decommissioning partly by inhibiting the mediator complex, which involves direct repression of mediator complex core components as well as indirect enhancement of the mediator complex inhibitor Med12.

### Syp promotes NB cell cycle exit by permitting nuclear Pros accumulation

**Fig. 6.**
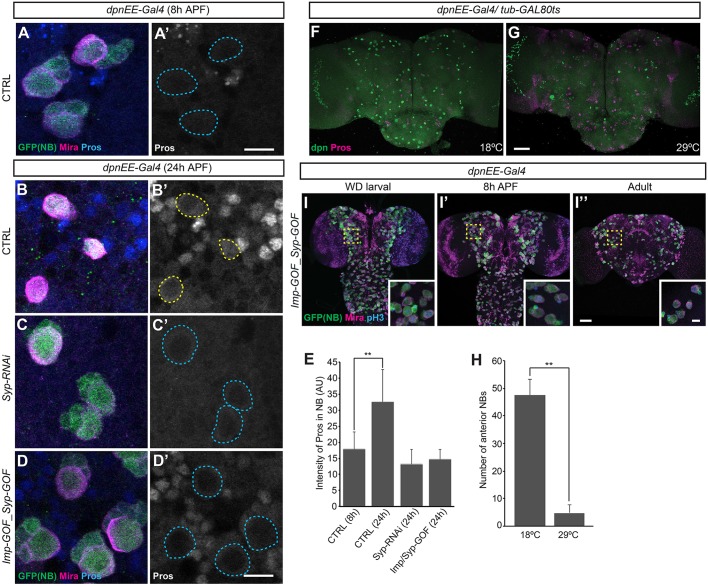
**Syp ensures NB exit by promoting Pros accumulation.** (A-D′) Syp is required for the accumulation of Pros. Representative confocal images of 8 h APF control (CTRL; A), and 24 h APF CTRL (B), *Syp* RNAi (C) and *Syp/Imp* gain-of-function (D) fly brains driven by *dpnEE-Gal4* and immunostained for GFP (green), Mira (magenta) and Pros (blue). The NBs are outlined with dashed lines. Scale bar: 10 µm. (E) Quantification of the grayscale value for NB Pros immunostaining as shown in A-D. **P<0.01 (Student's *t*-test) (mean±s.d., *n*=6 brains). (F,G) Pros induction terminated the long-lasting Syp-depleted neuroblasts. Composite confocal images of fly brains in which neuroblasts were depleted of Syp without (F) or with (G) Pros induction at early pupal development. Brains were immunostained for Dpn (green) and Pros (magenta). Scale bar: 50 µm. (H) Quantification of the number of neuroblasts (Dpn-positive cells) in fly brains (mean±s.d., *n*=6) as shown in F and G. **P<0.01 (Student's *t*-test). (I-I″) Ectopic Imp is dominant to Syp for NB decommissioning. Composite confocal images of Syp/Imp gain-of-function fly brains at specific developmental ages. Brains were immunostained for GFP (green), Mira (magenta) and phospho-Histone H3 (pH3, blue). Insets show the boxed areas at higher magnification. Scale bar: 50 µm (10 µm in inset).

Aged NBs with reduced cell size require Syp to exit the cell cycle (Fig. 1D, Fig. 5A-A‴). In wild-type NBs, nuclear accumulation of Pros promotes NB cell cycle exit (Maurange et al., 2008). Moreover, Syp protein may bind directly with *pros* mRNA as evidenced by a great enrichment of *pros* mRNA in Syp RIP (McDermott et al., 2014). These phenomena raised the possibility that Syp RNAi delays NB cell cycle exit by preventing Pros accumulation. Pros immunostaining in wild-type pupal brains revealed a drastic increase of Pros among the progeny of pupal NBs (Fig. 6B,B′ compared with 6A,A′). Knocking down Syp in NBs by targeted RNAi suppressed the enhancement of Pros in the late-born progeny (Fig. 6C,C′), apparently without affecting the basal Pros levels required to prevent dedifferentiation of GMCs. In late NBs, Syp RNAi significantly reduced the modest levels of Pros proteins and consistently excluded Pros from entering the nuclei (Fig. 6C,C′,E compared with 6B,B′). It has been shown that acute induction of transgenic Pros can promptly terminate actively cycling NBs (Li and Vaessin, 2000). We found transgenic Pros to be equally potent in the termination of otherwise long-lasting Syp-depleted NBs (Fig. 6F-H). These results indicate that Syp enhances Pros accumulation, which in turn promotes NB cell cycle exit.

Notably, Syp is required but not sufficient to increase Pros protein levels. For instance, a continuous induction of Syp from the beginning of neurogenesis failed to elicit precocious Pros accumulation in either NBs or their progeny (data not shown). Co-induction of Imp and Syp throughout neurogenesis did not accelerate or delay Pros expression among serially derived progeny, ascribing the increase of Pros in late-born neurons to temporal cues independent of Imp/Syp (Fig. 6D,D′). By contrast, ectopic Imp could effectively prevent Pros accumulation in Syp-positive NBs (Fig. 6D,D′,E,I-I″), arguing that activation of NB decommissioning is a prerequisite to nuclear accumulation of Pros. Consequently, we suggest that Syp plays a permissive role in the enhancement of Pros during late larval and early pupal neurogenesis.

In sum, Imp negatively regulates the ecdysone-induced, mediator complex-dependent decommissioning of NBs. The decommissioning triggers a timer for up-regulating *pros*; the ultimate increase in Pros protein requires Syp. This thereby allows individual NBs to have independent decommissioning programs based on temporal progression of Imp/Syp gradients.

## DISCUSSION

Decommissioning of NBs is a temporally controlled developmental program that proceeds from NB shrinking to cell cycle exit. Knoblich's group has shown that the mediator complex acts around pupation to uncouple the cell cycle from cell growth (Homem et al., 2014). This accompanies a change in cellular metabolism, which shrinks all but MB NBs. It is also known that a subsequent nuclear accumulation of Pros protein results in NB termination via a symmetrical division into two post-mitotic cells (Maurange et al., 2008). Here, we show that NB shrinking and cell cycle exit can be dissociated. In particular, enhancing mediator complex function causes Syp-depleted NBs to shrink, but not end (Fig. 5A-A″″). The long-lasting tiny NBs can proliferate even in adult brains (Fig. 1D″″, Fig. 5A″″, Fig. S1). This phenomenon indicates that the mediator complex is selectively involved in NB shrinkage, and that NB shrinkage does not automatically lead to cell cycle exit.

Imp can suppress NB shrinkage and in turn prevent cell cycle exit (Fig. 1C-C″″, Fig. 2A,A′, Fig. 3, Fig. 6). By contrast, Syp is selectively required for NB cell cycle exit (Fig. 1B-B″″, Fig. 5A-A″″, Fig. 6). We established their respective roles by directly controlling both Imp and Syp to mitigate the fact that they negatively regulate each other (Liu et al., 2015). Silencing Imp made the ‘forever-young’ Syp-depleted NBs shrink rapidly in early pupae but only moderately reduced the number of NBs ectopically present in adult brains (Fig. 1D-D″″, Fig. S1C). This ascribes the suppression of NB shrinkage in Syp-depleted NBs to an increase in Imp rather than reduced Syp (Fig. 1B-B″″,F). It further demonstrates the requirement of Syp for NB cell cycle exit.

Ectopic Imp can override the Syp-dependent cell cycle exit, as overexpressing Imp not only blocked NB shrinkage but also prevented cell cycle exit even in the presence of enhanced Syp (Fig. 1C-C″″,G, Fig. 6I-I″). The dominant role of Imp effectively explains why MB NBs, which have prolonged Imp expression, can escape the global initiation of NB decommissioning by ecdysone signaling. Consistent with this idea, repressing Imp, but not overexpressing Syp, made MB NBs shrink rapidly in early pupae (Fig. 2). The MB NBs with reduced cell size showed no evidence of cycling but could survive into late pupal stage despite strong Syp expression. This is consistent with normal ending of MB NBs by apoptosis rather than through Syp-dependent, Pros-mediated cell cycle exit (Siegrist et al., 2010). Insulin signaling and the transcription factor Retinal homeobox are known to promote MB NB proliferation and survival (Kraft et al., 2016; Lin et al., 2013; Siegrist et al., 2010). Although endogenous Imp protects MB NBs from shrinking in early pupae through inhibiting the mediator complex, it is unclear how ectopic Imp can extend MB NBs into adults that might involve different mechanisms.

Late larval induction of transgenic Imp could protect the normally ‘aged’ NBs from decommissioning in early pupae. However, a slightly later induction around pupation failed to show any protection (Fig. 3C). This assigns the critical window of Imp's action to be at or before the prepupal ecdysone pulse. The nearness of the window to the start of decommissioning suggests that the Imp mRNA-binding protein itself (rather than the accumulative effects of protracted Imp expression) suppresses the initiation of NB decommissioning by ecdysone. Being able to protect most NBs by acute Imp induction further implies that NBs do not age chronically as a result of repeated cycling.

Knocking down Med12 to enhance the mediator complex's function could largely erase the negative effects of ectopic Imp on NB shrinkage and cell cycle exit (Fig. 5B-B″″). However, overexpressing Med12 alone only partially blocks the rapid shrinkage of the Imp-depleted MB NBs (Fig. 5D). This argues that prolonged Imp expression protects MB NBs from early pupal decommissioning not only by enhancing Med12 but also by regulating other effectors to suppress the mediator complex. Consistent with this notion, we found that the mRNAs of several mediator complex core components, including *Med6/27/31*, can be greatly enriched by Imp RIP, implicating them as Imp direct targets (Fig. 4I). By contrast, Imp RIP failed to pull down *Med12* mRNA, suggesting an indirect positive regulation of Med12 by Imp. Besides, high Imp blocked NB decommissioning to a much larger degree than did inhibiting the mediator complex, suggesting possible involvement of mediator complex-independent targets. Imp could regulate a number of targets through diverse mechanisms, including localization, stability, translation, and nuclear export (Degrauwe et al., 2016). For example, Imp can promote the survival of target transcripts by counteracting the actions of siRNA (Toledano et al., 2012). Taken together, our data indicate that Imp could regulate the mediator complex and other ecdysone downstream effectors by controlling mRNA stability and/or translation to prevent NBs from decommissioning until the production of most progeny.

With the exception of the MB, aged NBs exit the cell cycle due to nuclear accumulation of Pros. Drastic enhancement of Pros levels also occurs among the newborn progeny from late larval and early pupal NBs. Syp is required for the nuclear accumulation of Pros in aged NBs as well as the strong Pros expression in late-born neurons (Fig. 6). However, strong Syp alone is not sufficient to increase Pros protein levels, even though Syp protein might bind directly with *pros* mRNA to enhance its stability and/or translation (McDermott et al., 2014). These observations argue that Pros is regulated at both transcriptional and post-transcriptional levels. As to timely cell cycle exit, a Syp-independent mechanism may upregulate *pros* transcription at certain times following the activation of NB decommissioning. Strong Syp levels then allow prompt increases in Pros proteins, leading to nuclear accumulation of Pros and termination of the stem cell mode of asymmetric cell division.

**Fig. 7.**
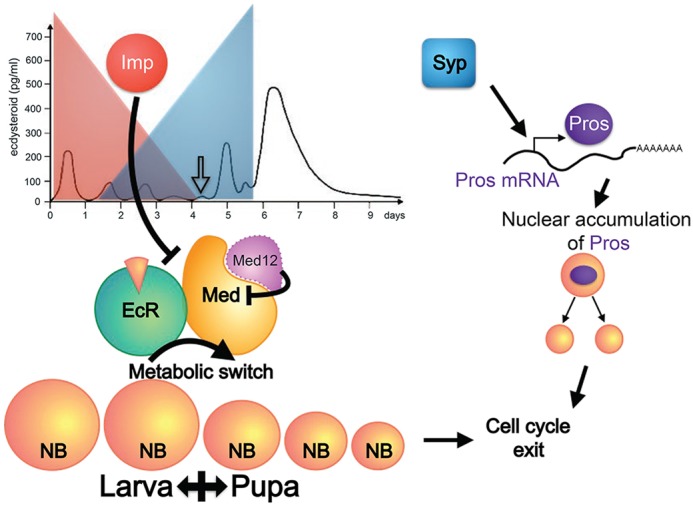
**Regulation of NB decommissioning by Imp/Syp RNA-binding proteins.** Reduction of Imp prior to the ecdysone signaling during metamorphosis (indicated by the unfilled arrow) is permissive for the ecdysone receptor (EcR)- and mediator complex-mediated metabolic switch that triggers non-MB NB decommissioning. Syp then promotes Pros accumulation to terminate NB cycling by terminal differentiation.

Imp/Syp levels in post-mitotic cells determine their birth time/order-dependent cell fates (Liu et al., 2015; Ren et al., 2017). Imp/Syp expression levels in progenitors govern their readiness for ageing and cell cycle exit. This allows decommissioning to be selectively activated in NBs when they have produced essential progeny and are ready for ageing. The opposing Imp/Syp temporal gradients with distinct lineage-specific temporal dynamics could therefore tailor neurogenic programs characteristic of different neuronal lineages (Fig. 7). Notably, various stem cells across diverse species express analogous descending Imp gradients (Nishino, et al., 2013; Toledano, et al., 2012). It is possible that the mechanisms of progeny temporal fating and progenitor ageing co-evolved on the pre-existing Imp gradients in neural stem cells. We propose that co-regulation of progeny temporal fate and progenitor decommissioning achieves the complex protracted neuronal lineages required for building sophisticated brains.

## MATERIALS AND METHODS

### Fly strains and DNA constructs

The fly strains used in this study include: (1) *dpnEE-Gal4* (Awasaki et al., 2014; Yang et al., 2016); (2) *UAS-Syp-RNAi* [stock #33011 and 33012, Vienna *Drosophila* Resource Center (VDRC, www.vdrc.at)]; (3) *UAS-Imp-RNAi* (stock #34977 and 55645, Bloomington *Drosophila* Stock Center); (4) *UAS-Imp-RM-Flag* (Liu et al., 2015); (5) *UAS-Syp-RB-HA* (Liu et al., 2015); (6) *UAS-Med12-RNAi* (stock #34588, Bloomington *Drosophila* Stock Center); (7) *UAS-pros.S* (stock#32245, Bloomington *Drosophila* Stock Center); (8) *UAS-Med6-RNAi* (stock #33743, Bloomington *Drosophila* Stock Center); (9) *lexAop-Syp-miRNA* (Ren et al., 2017); (10) *UAS-Kto.J* (stock #63801, Bloomington *Drosophila* Stock Center); (11) *FRTG13, UAS-mCD8::GFP; GAL4-OK107* (Lee et al., 1999); (12) *Imp[CB04573]* (gift from D. L. Jones, The Salk Institute for Biological Studies, CA, USA; Toledano et al., 2012); and (13) *UAS-p35* (Hay et al., 1994). The following strains were generated in this study: (14) *UAS-GFP::Sec*; (15) *dpn-LexA::P65*; and (16) *act5C-GAL80^ts^-insulated spacer-tub-GAL80^ts^*.

For *UAS-GFP::sec*, the GFP fragment was inserted in a *Not*I/*Xba*I site in *5xUAS-pMUH* plasmid (Awasaki et al., 2014), then the N-terminal 1-50 amino acids of securin was inserted in-frame at the C terminus of GFP in an *Eco*RI/*Xho*I site.

For *dpn-LexA::P65*, a previously characterized *dpn* NB enhancer (Emery and Bier, 1995) and a *Drosophila* synthetic core promoter (Pfeiffer et al., 2008) were inserted in front of *LexA::P65* in *pBPLexA::P65w* (Pfeiffer et al., 2010) by gateway cloning (Invitrogen).

For *act5C-GAL80^ts^-insulated spacer-tub-GAL80^ts^*, a *pGAL80^ts^* plasmid was created by removing the *Hin*dIII/*Bgl*II fragment in *pJFRC-MUH* (Pfeiffer et al., 2010) then inserting the GLA80^ts^ fragment from *pMK-RQ(KanR)-GAL80^ts^* into a *Kpn*I/*Xba*I site. The *tubulin* promoter was inserted in front of GAL80^ts^ in *pGAL80^ts^* through gateway cloning. The *Actin 5C* promoter was inserted in front of GAL80^ts^ in *pGAL80ts* by gateway cloning. A synthetic insulated spacer cassette (Pfeiffer et al., 2010) was inserted at a *Fse*I site in *pAct5C-GAL80^ts^*, and the *Eco*RI fragment of *pTub-GAL80^ts^* was then inserted in the *Eco*RI site after the insulated spacer cassette.

### Temporal induction of Pros

Flies with genotype *UAS-pros.S; act5C-GAL80^ts^-insulated spacer-tub-GAL80^ts^/dpn-LexA::P65; 13XlexAop-Syp-miRNA/dpnEE-Gal4* were cultured at 18°C. The GAL80 and non-temperature-sensitive LexA::p65 allowed Syp knockdown continuously. For the control experiment, collected white pupae were cultured at 18°C for 24 h before dissection. To induce ectopic Pros in NBs, collected white pupae were transferred to 29°C to inactivate the temperature sensitive GAL80 for 16 h before dissection and thus de-repress *dpnEE-Gal4* for misexpression of Pros.

### Temporal induction of Imp

Embryos with genotype *act5C-GAL80^ts^-insulated spacer-tub-GAL80^ts^/dpnEE-Gal4; UAS-Imp-RM-Flag/UAS-GFP::Sec* were collected for 24 h at 18°C and then cultured at 18°C for 5 days. The larvae were heat shocked at 37°C for 15 min and incubated at 29°C to inactivate the temperature-sensitive GAL80. White pupae were then collected at several time points (0, 8, 12, 15, 18 and 24 h) after heat shock, which resulted in the induction of Imp expression at different developmental time points (0, 8, 12, 15, 18 and 24 h) before pupal formation (BPF). Collected white pupae were cultured at 29°C and dissected at 38 h APF.

### Immunohistochemistry and confocal imaging

Brain tissues at specific developmental stages were dissected and immunostained as described previously (Lin et al., 2012). The following primary antibodies were used: chicken anti-GFP (1:1000, A10262, Life Technologies), rat anti-Mira (1:200, ab197788, abcam; shows non-specific signals in central complex neuropil), rat anti-Dpn (1:200, ab195172, abcam), rabbit anti-pH3 (1:500, #9701, Cell Signaling), mouse anti-Pros (1:200, Developmental Studies Hybridoma Bank), guinea pig anti-Med12 (1:500, gift from J. Treisman, NYU School of Medicine, NY, USA; Janody, et al., 2003), rabbit anti-Imp (1:600, gift from P. Macdonald, University of Texas at Austin, TX, USA; Geng and Macdonald, 2006) and guinea pig anti-Syp (1:500; McDermott et al., 2014). All corresponding fluorescent secondary antibodies (1:500) were purchased from Life Technologies and confocal images of whole-mount fly brains were collected on a Zeiss LSM 710 confocal microscope.

### Image analysis

To measure Imp signal intensity in the MB (Fig. 2), all NBs were labeled with *dpnEE>GFP::Sec* and MB NBs were determined based on high Imp expression in the progenies. We selectively analyzed those NBs (circled) with a maximum diameter at the chosen focal planes compared with neighboring focal planes. Confocal images were exported to Adobe Photoshop, a hand-drawn mask was created for the cytoplasmic region of selected NBs (circled in Fig. 2), and the average intensity of grayscale value for each pre-defined region was calculated using the ‘Histograms’ algorithm in Photoshop. The grayscale value in the nuclear region was used to normalize the intensity across different sections/samples.

To compare Med12 (Fig. 4), Pros (Fig. 6) and Imp/Syp (Fig. S2) levels across various genotypes, images were taken using the same confocal setting (pinhole size, gain, laser power, etc.) and an image of selected focal plane was exported to Adobe Photoshop. A hand-drawn mask was created for the cytoplasmic (for Imp/Syp) or nuclear (for Med12 and Pros) region of the cell of interest with a maximum diameter at selected focal plane. The averaged grayscale value for each pre-defined region was calculated using the ‘Histograms’ algorithm in Photoshop. Relative intensity for Med12 is the ratio of averaged grayscale value in central brain to that of optic lobe NBs. The grayscale values of Pros and Imp/Syp were normalized to the background staining in the developing optic lobe.

### Immunoprecipitation

Third instar larval brains dissected in Schneider medium were homogenized in 150 µl immunoprecipitation (IP) buffer [50 mM Tris-HCl pH 8.0, 150 mM NaCl, 0.5% NP-40, 10% glycerol, complete EDTA-free protease inhibitor and RNase inhibitor (RNAsin Plus RNase Inhibitor, Promega)]. The lysate was precleared with 45 µl of washed Pierce Control Agarose Resin (Thermo Fisher Scientific). For each reaction, 50 µl of pre-cleared lysate was taken as a 50% input sample directly to RNA extraction. Next, 100 µl of pre-cleared lysate was incubated with 25 µl of GFP-TRAP agarose beads (Chromotek) for 2 h at 4°C with rotation. The beads were washed four times briefly each with 200 µl cold IP buffer at 4°C. After the final wash, beads were re-suspended in 100 µl extraction buffer (50 mM Tris-HCl pH 8.0, 10 mM EDTA and 1.3% SDS, 1:100 RNAsin) and incubated at 65°C, 1000 rpm (mixing frequency) for 30 min in a thermomixer. The elution step was repeated and the supernatants were pooled. RNA was extracted from inputs and immunoprecipitates with the RNAspin RNA Isolation kit (GE Healthcare) and eluted in 40 µl of nuclease-free H_2_O. Reverse transcription was performed using RevertAid Premium Reverse Transcriptase (Thermo Fisher Scientific) with random hexamer primers. cDNA was then used as a template for real-time quantitative PCR.

### Real-time quantitative PCR (RT-qPCR)

RT-qPCR was performed with SYBRGreen Mastermix (Thermo Fisher Scientific) using a real-time PCR detection system [CFX96 TouchTM Real-Time PCR Detection System (Bio-Rad)]. Cycle threshold [C(_T_)] value was calculated by the Bio-Rad CFX software using a second differential maximum method. A dilution series of the input sample allowed the percentage input of each gene to be calculated to assess immunoprecipitation efficiency. The forward and reverse qPCR primer sequences are listed in Table S1.
